# An in vivo immunomodulatory and anti-inflammatory study of fermented *Dendropanax morbifera* Léveille leaf extract

**DOI:** 10.1186/s12906-018-2282-x

**Published:** 2018-07-24

**Authors:** Biruk Tesfaye Birhanu, Jin-Yoon Kim, Md. Akil Hossain, Jae-Won Choi, Sam-Pin Lee, Seung-Chun Park

**Affiliations:** 1 Laboratory of Veterinary Pharmacokinetics and Pharmacodynamics, College of Veterinary Medicine, Kyungpook National University, Bukgu, 80 Daehakro, Daegu, 41566 South Korea; 2 Veterinary drugs & Biologics Division, Animal and Plant Quarantine Agency (QIA), 177, Hyeoksin 8-ro, Gimcheon-si, Gyeongsangbuk-do 39660 South Korea; 3 The Center for Traditional Microorganism Resources (TMR), Keimyung University, Daegu, 704-701 South Korea

**Keywords:** *Dendropanax morbifera* Léveille, Immunomodulation, T-cell proliferation, Proinflammatory cytokines

## Abstract

**Background:**

Medicinal plants represent a source of new drugs for the prevention and treatment of infectious diseases. *Dendropanax morbifera* Léveille is an economically and medicinally important subtropical tree that has various biological activities. However, its ability to affect immune responses in vivo is unknown. Hence, this study was designed to examine the immunomodulatory activity of fermented *D. morbifera* extract in BALB/c mice.

**Methods:**

five-week-old female BALB/c mice were arranged in six groups and kept under a standard laboratory condition. Splenocyte counts were determined using the trypan blue dye exclusion method, and splenic lymphocyte proliferation was determined using concanavalin A and lipopolysaccharide (LPS). Flow cytometric analysis was performed to phenotype T-lymphocytes. Next, cytokine and immunoglobulin quantitation was performed using sandwich ELISA.

**Results:**

The results showed an increase in spleen cells by 71 and 67% in mice treated with 125 and 250 mg/kg of *D. morbifera,* respectively. In addition, splenocyte proliferation was increased 58.7% in response to concanavalin A treatment, while LPS treatment induced a 73.3% increase in mice treated with 125 mg/kg. T-cell phenotypic analysis indicated that *D. morbifera*-treated groups showed higher CD8a+, CD11b and CD3+ T-cell expression. However, the treatment groups showed suppression of IL-1α, Il-1β and IL-4. In addition, the IgG super-family was downregulated in a dose-dependent manner by 4.5% up to 43.7%.

**Conclusions:**

Taken together, we show that *D. morbifera* increases the number and proliferation of T- and B-lymphocytes. Moreover, these effects may play a role in boosting non-specific immunity, while suppressing proinflammatory cytokines and immunoglobulins after a single antigen exposure.

## Background

Medicinal plants play a vital role in the treatment of human and animal diseases. The application of these herbal medicines has contributed significantly to the search for a new drug for prevention and treatment of infectious agents. Recently, much interest has been directed to the use of natural compounds to enhance host immunity.

Plant extracts play a significant role in the prevention and curing of infections by modulating the immune system. As a result, their application and use has increased dramatically [1]. Herbal medicines act on the immune system by either suppressing or stimulating innate or adaptive immune cells/molecules [2]. Immune regulation is important in maintaining normal immunity, and the search for herbal immunomodulatory compounds to treat various infections by enhancing the body’s natural resistance is of growing interest [3].

*Dendropanax morbifera* Léveille, also knowns as *Dendropanax trifidus,* is an economically and medicinally important subtropical broad-leaved tree that is endemic to Korea [4, 5]. The tree has been used in the treatment of different human infections and reported to have anti-thrombotic, anti-diabetic and anti-atherogenic components [6–9]. Polyacetylene from plant leaves has been shown to have an anti-complement effect [10]. The plant is also known to increase the excretion of toxic elements, namely, cadmium from the kidney, and to reduce cadmium-induced oxidative stress in the hippocampus [11]. In addition, Hyun and his colleagues [4] reported the anti-cancer and anti-oxidant activity of the methanolic leaf and debarked stem extracts. The bioflavonoid extract, rutin, prevents rotenone-induced cell injury through inhibition of the JNK and p38 MAPK signaling pathways in a Parkinson’s disease model [12]. Furthermore, the chloroform extract suppresses proinflammatory mediators and cytokines through inhibition of NF-κB [13].

Numerous studies have been conducted using fermented plant extracts with different techniques [14]. In our previous works [15], we optimized co-production of poly-γ-glutamic acid (γ-PGA) and γ-aminobutyric acid (GABA) in the presence of sodium-L-glutamate (MSG) in *Dendropanax morbifera* fermented by *Bacillus subtilis* HA (KCCM 10775P, patent strain) and *Lactobacillus plantarum* EJ2014 (KCCM 11545P, patent strain). The extract has shown to have an immunostimulatory activity in RAW 264.7 cells in in vitro experiment. A voucher of the specimen for the fermented extract was deposited at Keimyung Traditional center, Keimyung University.

To our knowledge, no studies have investigated the immunomodulatory activities of the fermented plant extract in vivo. Hence, the objective of this study was to determine the immunomodulatory activities of fermented *Dendropanax morbifera* Léveille leaf extract in BALB/c mice.

## Methods

### *Dendropanax morbifera* leaf extract preparation

*Dendropanax morbifera* leaf (Hambakjae Bio Farm Co., Ltd., Jeju Island, South Korea) was dried and 10 volume of water was added before macerated for 8 h at 98 °C. Fifty milliliter of the concentrated extract was supplemented with 5% (*v*/v) *Bacillus subtilis* and mixed with 3% monosodium glutamate (MSG) and 3% glucose and incubated for 3 days in shaking incubator with 160 rpm (SI-900R, Jeio Tech. Co., Ltd., Daejeon, Korea) at 42 °C for γ-PGA production. The first fermented product was mixed with *Lactobacillus plantarum* EJ2014 starter 1% (v/v) and incubated at 30 °C for 5 days in a constant temperature incubator (IS-971R, JeioTech, Kimpo, Korea) for GABA production through lactic acid fermentation. Finally, an additional 20% MSG, 20% glucose and 10% skim milk was supplemented to the solution and make the final volume 130 mL.

### Animals and study design

All experimental protocols involving animals were approved by the Kyungpook National University Ethical Committee (KNU 2016–121). Five-week-old female specific pathogen free (SPF) BALB/c mice were procured (ORIENT BIO, Republic of Korea) and acclimated to lab conditions for one week before the start of the study. The weight of the mice ranged from 18 to 20 g. The animal room was maintained at a relative humidity of 50–65% at 20 °C–24 °C temperature and equal 12 h light and dark time. Mice were provided with standard pellet diet and ad libitum filtered tap water access. After adaptation to laboratory conditions, the mice were arbitrarily divided into six groups, each consisting of ten mice (Table 1). The total number of mice used in the present study was calculated by the G*power program (3.1.9.2) based on effect size (0.5), α error probability (0.05), Power (1-β error probability) (0.8) and number of groups (6).

**Table 1:** Group arrangement and experimental design of mice

Groups	Treatment groups	Description
Group I	Normal control	Received only diet and water
Group II	Vehicle control	Received water and treated with SRBC
Group III	Positive control	Received Ginseng extract 200 mg/kg
Group IV	Low dosage treatment	Received 125 mg/kg of *D. morbifera*
Group V	Intermediate dosage treatment	Received 250 mg/kg of *D. morbifera*
Group VI	High dosage treatment	Received 500 mg/kg of *D. morbifera*

**Fig. 1:**
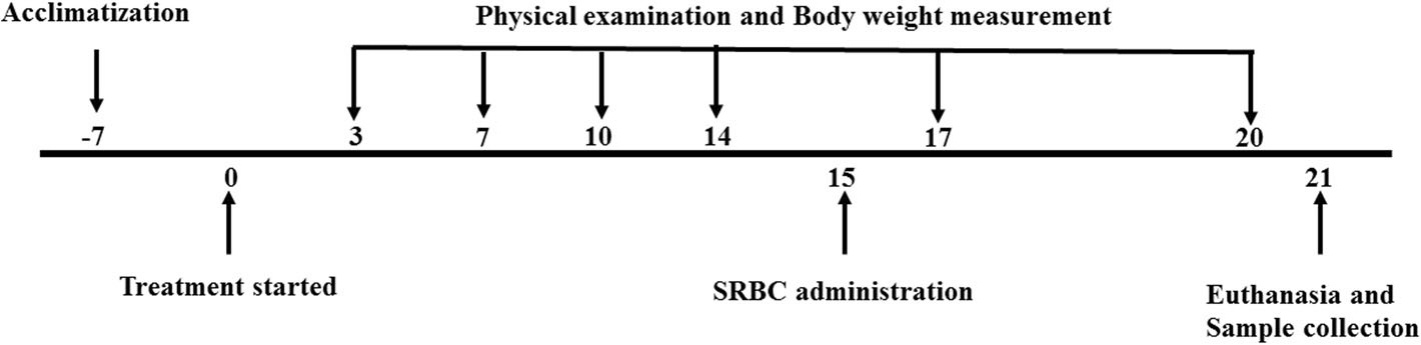
Flow chart of the experimental procedure

All mice in the treatment group received a daily oral dose of 200 μL of (125 mg/kg, 250 mg/kg and 500 mg/kg) *D. morbifera* extract for 14 days. The mice were immunized intraperitoneally with 5.0 × 10^8^ sheep red blood cells (SRBCs) per milliliter on the 15th day, and treatment continued with the extract for an additional week. Body weight was measured regularly to adjust dosing volumes. After 21 days, mice were euthanized by carbon dioxide inhalation. Euthanasia lid was placed over the cage. CO_2_ was delivered from the tank at a flow rate of 10–30% of the chamber per minute. Finally, animals were monitored for cessation of respiration and left in the chamber for at least 1 min after respiration has ceased, and blood and organs were collected (Fig. 1). Blood was collected in heparinized tubes and centrifuged at 10,000 RPM for 5 min. Plasma was separated and stored at − 70 °C until use in an ELISA. Moreover, the organs were weighed and processed accordingly.

### Acute toxicity test

The test was carried out on six 5-week-old female rats with an average weight of 139 g. The rats were fasted overnight prior to dosing. A single administration of the fermented *Dendropanax morbifera* Léveille plant extract was given orally to 3 rats at a dose of 2000 mg/kg. In addition, 3 rats were left untreated to serve as negative controls. Rats were checked for physical and clinical symptoms. Body weight and abnormal behavior, if observed, were recorded on a daily basis. In addition, pathological examination was performed after 14 days of observation. Rats were euthanized by carbon dioxide inhalation. Euthanasia lid was placed over the cage. CO_2_ was delivered from the tank at a flow rate of 10–30% of the chamber per minute. Finally, animals were monitored for cessation of respiration and left in the chamber for at least 1 min after respiration has ceased.

### Preparation of splenocytes

Splenocyte preparation was performed as previously described (Ahmad et al., 2015). Briefly, after sacrifice, mouse spleen and other organs were removed aseptically from all experimental groups. The tissues were placed into a sterile tube with Hank’s balanced salt solution (HBSS, Gibco Life Technologies, NY) on ice. The spleen was minced and pressed through a 70 μm fine nylon cell strainer (BD Biosciences, CA) using plunger at the end of a 3 mL syringe. The cells were washed with excess HBSS using the strainer. The cell suspensions were then centrifuged at 1600 rpm for 5 min. The supernatant was discarded, and the pellet was re-suspended in 1 mL pre-warmed Red Blood Cell Lysing Buffer solution (Sigma, UK). The cells were incubated at 37 °C for 1 min to lyse the RBCs before cold HBSS was added. Next, the cells were centrifuged for 5 min at 1600 rpm at 4 °C. The pelleted cells were washed three times with PBS after centrifugation. Afterwards, the supernatant was aspirated, and the pellet was re-suspended in RPMI-1640 medium (Sigma-Aldrich, MO) supplemented with 10% (*v*/v) FBS, 100 units/mL of penicillin and 100 μg/mL of streptomycin. The cell count was measured with a hemocytometer using the trypan blue dye exclusion method.

### Splenic lymphocyte proliferation assay

Spleen cells (4 × 10^6^ cells/mL) from the control and treatment groups were seeded (200 μL/well) into a 96-well plate in RPMI-1640 medium (10% FBS, 1% P/S). Cells from each group were cultured in the absence of any mitogen and in the presence of concanavalin A (5 μg/mL) or LPS (10 μg/mL) at 37 °C for 72 h. Results were read using an MTT (3-(4,5-Dimethylthiazol-2-yl)-2,5-Diphenyltetrazolium Bromide) reagent assay. The proliferation index was calculated by dividing the sample OD readings to the control OD value.

$$ \Pr \mathrm{oliferation}\kern0.17em \mathrm{index}=\frac{\mathrm{OD}\;\mathrm{of}\kern0.17em \mathrm{treated}\kern0.17em \mathrm{cells}}{\mathrm{OD}\;\mathrm{of}\kern0.17em \mathrm{control}\kern0.17em \mathrm{cells}} $$

### Flow cytometric analysis for T-lymphocyte phenotyping

For T-lymphocyte phenotyping of spleen cells (1 × 10^6^ cells/100 μL), they were harvested into FACS tubes. The cells were blocked with IgG blocking solution (1 μg IgG/10^6^ cells) for 15 min at room temperature. The conjugating antibodies (10 μL/10^6^ cells) were added and vortexed before the cells were incubated for 30 min in the dark. The cells were washed with Flow Cytometry Staining Buffer to remove the unbound antibody, and then suspended cells were centrifuged at 300 x g for 5 min. The cells were washed and re-suspended with 400 μL of the buffer solution for flow cytometric analysis. Finally, data were acquired and analyzed using multicolor FACS with FACSDiva version 6.1.3 software on BD FACSAria™ III (BD Biosciences, CA, US). Group comparisons were made among the different T-lymphocyte sub-populations, and the results were expressed as a percentage of expression.

### Cytokine and immunoglobulin determination

Samples, standards and controls were incubated for 2 h (cytokines) and 30 min (immunoglobulins) at room temperature on a horizontal orbital microplate shaker after 50 μL of the microparticle cocktail was added to each well of the microplate, and 50 μL of the standard or sample was added to each well. After washing four times with a magnetic device, 50 μL of diluted Biotin Antibody Cocktail was added to each well for the cytokines and immunoglobulins and incubated for 1.5 h at room temperature on the shaker. Diluted Streptavidin-PE (50 μL) was added to each well and incubated for 30 or 15 min at room temperature on the shaker after washing. Finally, the microparticles were resuspended by adding 100 μL of Wash Buffer to each well and incubated for another 2 min before reading using a Luminex MAGPIX analyzer (Luminex, Austin, TX, USA). Each analyte in the sample reacted independently with the corresponding antibody attached to the specific number bead. The concentration of the samples was determined after a standard curve was generated using five parameters logistic curve-fit (MasterPlex QT 2010 (MiraiBio, Hitachi, CA, USA) and multiplied by the dilution factor.

### Statistical analysis

Data were analyzed using a one-way and two-way analysis of variance followed by Dunnett’s test and Tukey’s test using GraphPad Prism 7 software. The results are expressed as the mean ± standard error of the mean. *P* < 0.05 was considered as statistically significant.

## Results

The animal experiment was carried out for 21 days, and mice were supplemented with pelleted feed and water ad libitum. The amount of feed and water was measured twice a week, and a total of six measurements was carried out. The feed and water intake of the animals were reduced immediately after treatment with SRBC and increased towards the end of treatment. Although the results showed that mice treated with *D. morbifera* Léveille had gained more weight than the untreated groups, there is no significant difference observed between the treated and control groups for water and food intake.

The acute toxicity result showed that there were no physical or clinical signs observed in the treated rats from day zero to day fourteen. In addition, no apparent pathological lesions were found in the organs of the mice that received the treatment, and no mortality of rats was recorded.

**Fig. 2:**
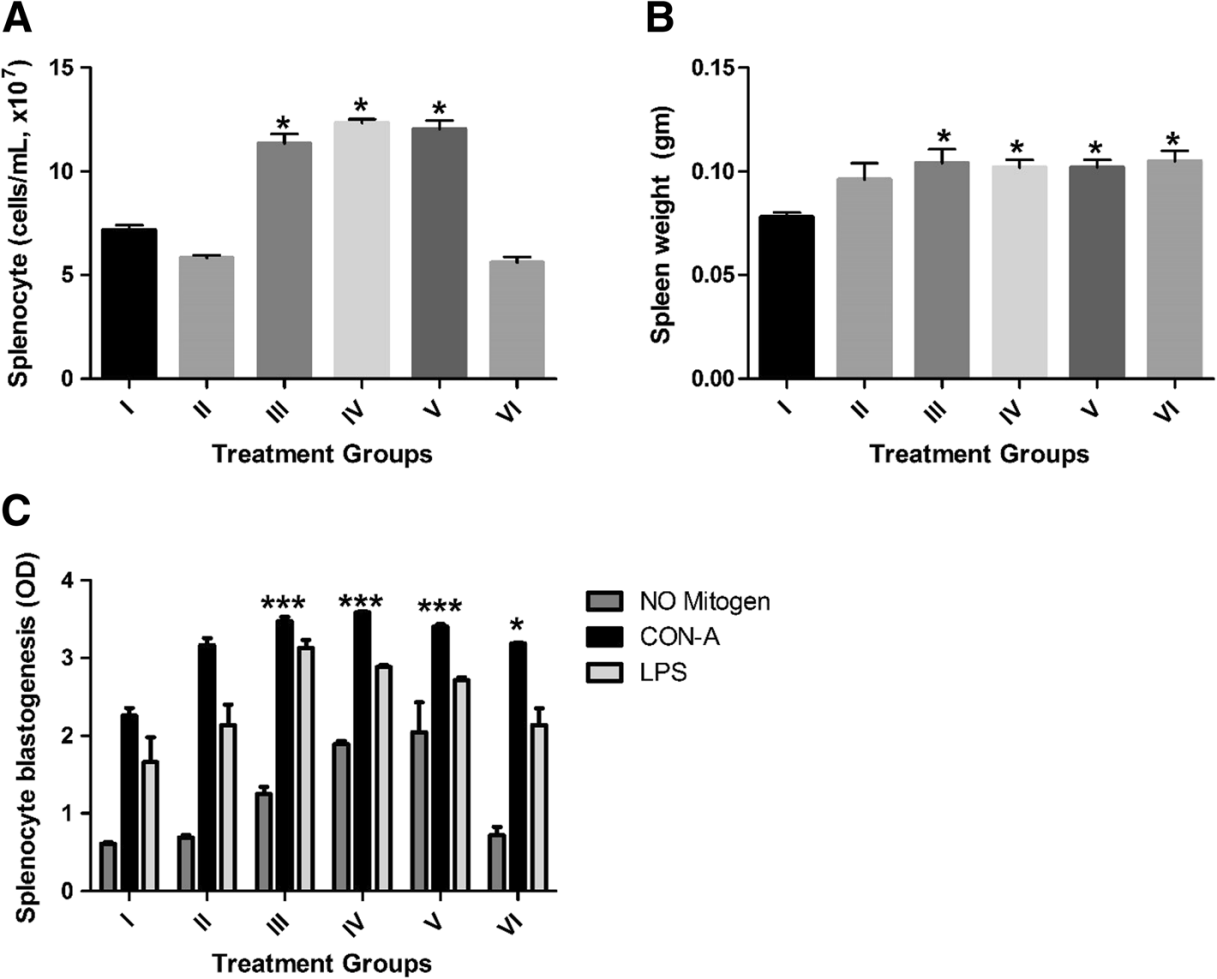
Effect of *Denropanax morbifera* on the splenocytes of mice. **a** Total splenocyte count of different treatment groups. A significant increase in splenocyte counts was observed in mice among control groups and those that received ginseng extract or 125 and 250 mg/kg of *D. morbifera* Léveille. **b** Weight of spleen taken from each mouse group. **c** In vivo activation of splenocytes by Con A and LPS after 72 h of incubation. A significant difference was observed between group II and group III, as well as groups V and V treated with Con-A and LPS (*P value < 0.05; ***P < 0.001)

In addition, the spleen, liver, thymus and both kidneys from each mouse were removed aseptically and measured accordingly at the end of the experiment (Table 2, Fig. 2b). A significant difference in the weight increase of spleens from mice receiving the ginseng extract or 125, 250 or 500 mg/kg of *D. morbifera* Léveille was observed compared with the other groups (*P* < 0.05). The spleen weight increased by 33.3, 30.8, 20.5 and 34.6% in mice treated with ginseng extract or 125, 250 or 500 mg/kg of *D. morbifera,* respectively.

**Table 2:** The average weight of the organs collected from treated and non-treated mice

	Spleen (Mean ± SEM)	Liver (Mean ± SEM)	Thymus (Mean ± SEM)	Left Kidney (Mean ± SEM)	Right Kidney (Mean ± SEM)
I	0.08 ± 0.002	0.854 ± 0.022	0.03 ± 0.0084	0.106 ± 0.006	0.110 ± 0.005
II	0.096 ± 0.008	0.824 ± 0.035	0.038 ± 0.0049	0.122 ± 0.007	0.116 ± 0.008
III	0.104 ± 0.007	0.894 ± 0.038	0.047 ± 0.0037	0.114 ± 0.007	0.12 ± 0.005
IV	0.102 ± 0.004	0.878 ± 0.024	0.059 ± 0.0086	0.112 ± 0.004	0.116 ± 0.004
V	0.103 ± 0.005	0.84 ± 0.028	0.049 ± 0.0031	0.114 ± 0.005	0.112 ± 0.002
VI	0.105 ± 0.005	0.863 ± 0.013	0.047 ± 0.0034	0.115 ± 0.006	0.128 ± 0.005

*Weight presented in grams. SEM; standard error of the mean

Spleen cells were counted with a hemocytometer during the trypan Blue dye-exclusion assay. The total number of counted spleen cells was found to be increased in mice treated with the ginseng extract or 125 or 250 mg/kg of *D. morbifera* Léveille by 58, 71 and 67%, respectively (*P* < 0.001). However, spleen cell counts at the higher extract dosage of 500 mg/kg did not show any significant increase when compared with untreated control groups (Fig. 2a).

T- and B-cell lymphocyte proliferation assays were conducted using Con-A and LPS. Our in vivo and ex vivo (data not shown) results showed that splenocyte proliferation by con A and LPS treatment is four-fold higher than that of cells with no-mitogen activation (Fig. 2c). The OD value at 570 nm was significantly higher in mice treated with *D. morbifera* and in the positive control group for both the Con-A and LPS tests. Spleen cell proliferation after Con-A treatment was increased by 53.6, 58.7, 50.6 and 41% for mice treated with ginseng extract or 125, 250 or 500 mg/kg of *D. morbifera,* respectively. In addition, B cell proliferation by LPS was increased by 88.1, 73.3, 63.1 and 28% in mice treated with ginseng extract or 125, 250 or 500 mg/kg of *D. morbifera,* respectively.

**Fig. 3:**
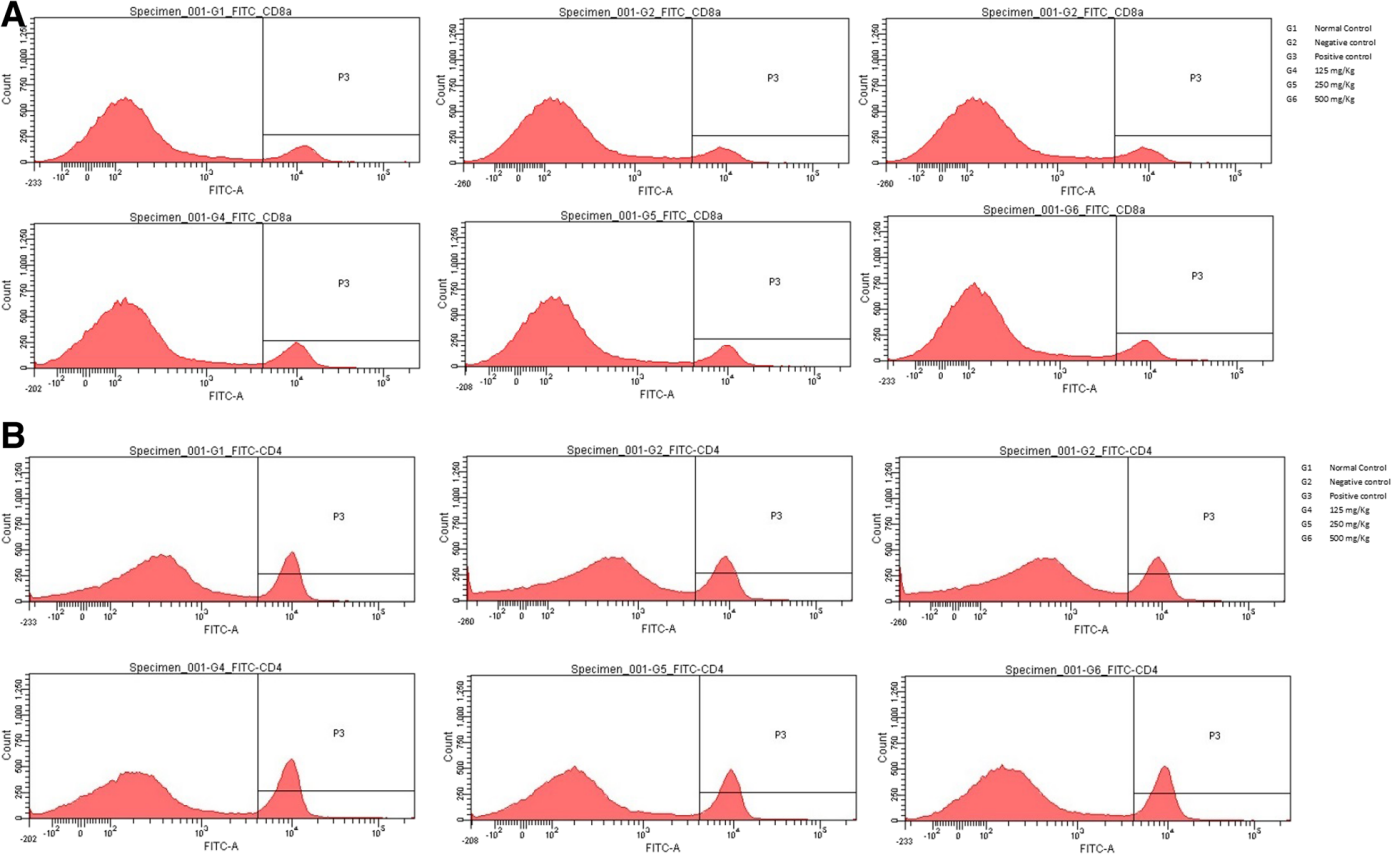
FITC-conjugated CD8a (**a**) and PE-conjugated CD3 (**b**) T cell population comparison among different treatment groups

Flow cytometry was performed for phenotyping the T-lymphocyte population. *D. morbifera* treated groups showed higher CD8a, CD3 and CD45RA T-cell expression than the control groups. The increase is significantly higher in those treated with 125 mg/kg of *D. morbifera* treatment, with percentages of 16.7, 5.7 and 7.1% for CD8a, CD3 and CD45RA T cells, respectively (Fig. 3). In addition, our results showed a dose-dependent reduction in the T-cell lymphocyte populations after the same treatment conditions as mentioned above (Fig. 3). Conversely, CD11b-expressing cells showed a significant reduction of 22% in low dosage (125 mg/kg)-treated mice.

**Fig. 4:**
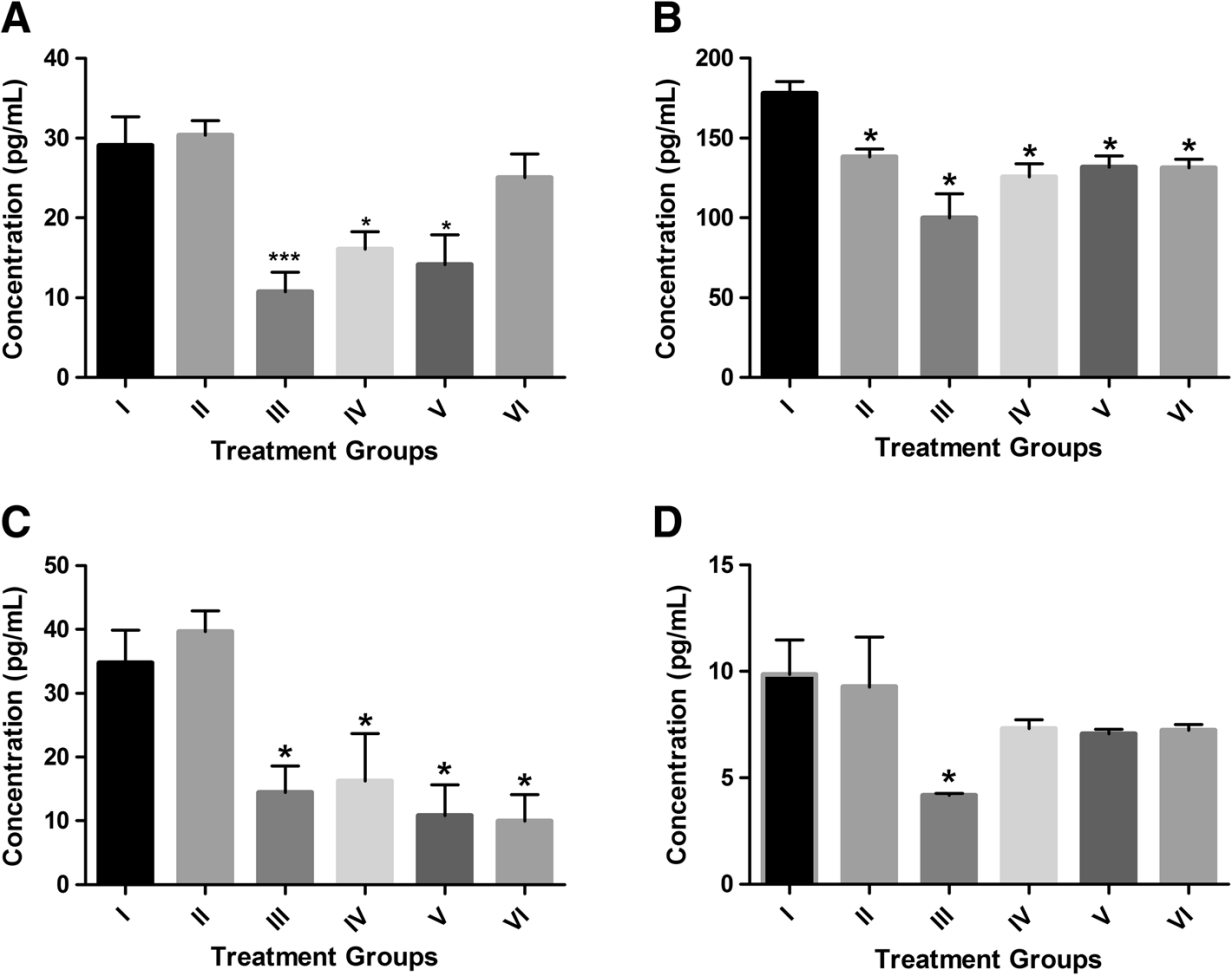
Cytokine concentration in different treatment groups of mice. **a** IL-1α, **b** IL-1β, **c** IL-4 and **d** IL-9. In the IL-1α test group III, IV and V, a significant reduction was observed when compared with group II mice. The IL-4 level was significantly reduced in groups III, V and VI when compared with group II mice. No significant difference was observed in IL-1β and IL-9 levels in any treatment groups

Sandwich ELISA was performed to determine the effects of *D. morbifera* activity on Th1- and Th2- dependent cytokine release and adaptive immunity. The level of IL-1α, IL-1β and IL-4 was suppressed in mice treated with ginseng and *D. morbifera* extract (Fig. 4). The level of IL-4 was reduced in a dose-dependent manner by 53.2, 68.9 and 71.4% in mice treated with 125, 250 and 500 mg/kg *D. morbifera*, respectively. However, the levels of TNF-α, IL-2, IL-5, IL-6, IL-10, IL-12, IL-12p70 and IL-13 were below the detection limit of the ELISA kit. IFN-γ levels were elevated upon treatment with increasing *D. morbifera* treatment, although a significant difference was not observed among the groups.

**Fig. 5:**
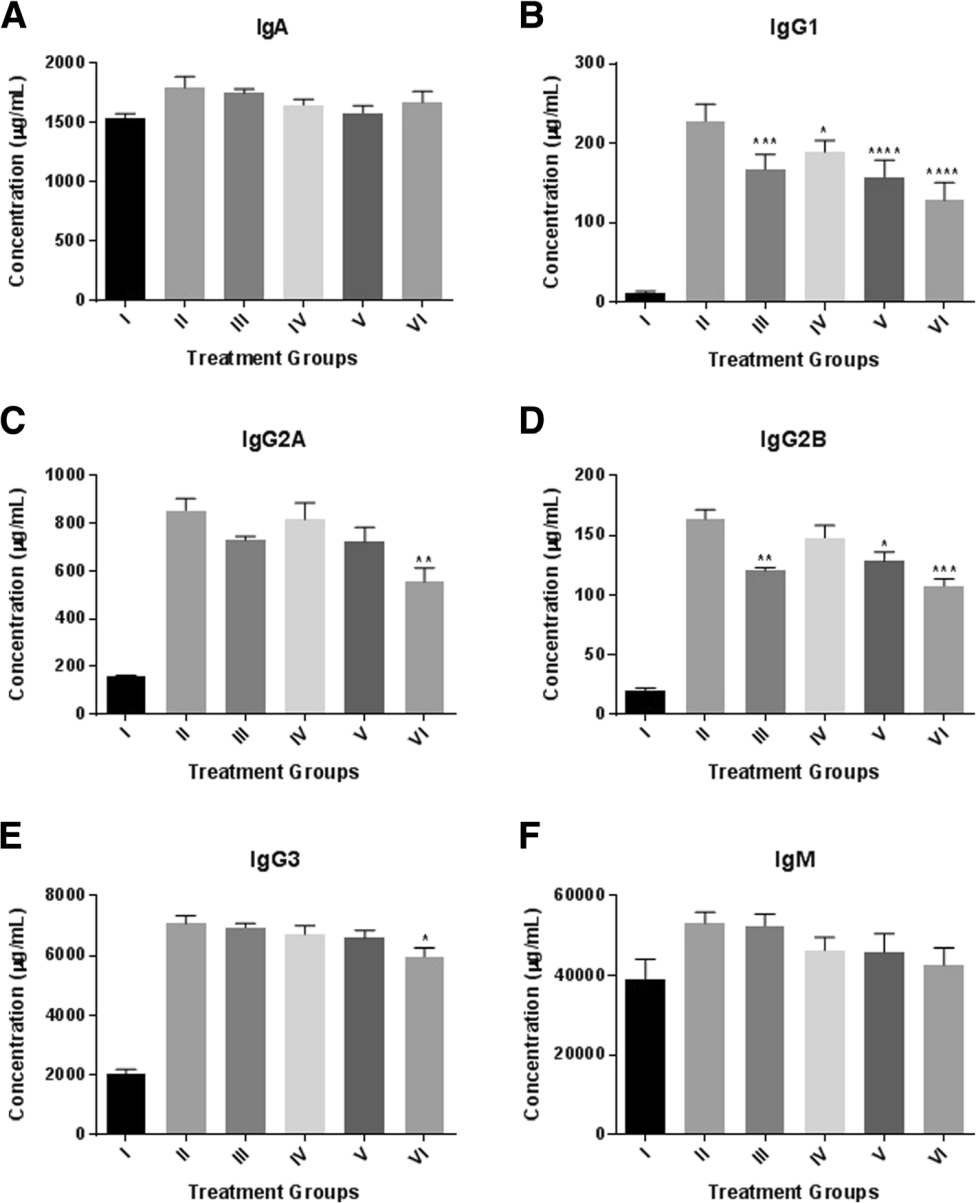
Immunoglobulin levels of control and *D. morbifera* Léveille-treated mice. **a** IgA, **b** IgG1, **c** IgG2A, **d** IgG2B, **e** IgG3 and **f** IgM. All the tested immunoglobulins showed a dose-dependent reduction in treatment with *D. moribifera* Léveille, except for IgA. Group VI showed a significant reduction IgG2A levels. IgG2B was significantly reduced in groups III, V and VI. IgG3 showed significant suppression in group VI mice. No significant difference was observed between the IgM treatment groups. (* = *P* value < 0.05; ** = *P* value < 0.01; *** = *P* value< 0.001; **** = *P* value < 0.0001; all the comparisons were made using the untreated control groups)

*D. morbifera* Léveille results in the suppression of the tested immunoglobulins IgA, IgG (IgG1, IgG2A, IgG2B IgG3) and IgM in a dose-dependent manner (Fig. 5). The IgG super-family was increased when compared with normal control groups. However, immunoglobulins were reduced by 17.1, 31 and 43.7% for IgG1; 4.5, 15.4 and 35% for IgG2A; 9.5, 21.5 and 34.3% for IgG2B and 5.1, 6.8 and 15.6% for IgG3 in mice treated with 125, 250 and 500 mg/kg of *D. morbifera* compared to the antigen receiving control group.

## Discussion

The immunomodulatory activity of plant extracts that stimulate or suppress the immune system, may be of paramount importance in regulating diseases that originate from immune cell disturbances. Moreover, plant extracts can provide a substitute for the currently available chemotherapies aimed at combating diseases such as autoimmune diseases. *D. morbifera* Léveille is a well-known Korean traditional medicine used for improvement of blood circulation that has anti-oxidant, anti-inflammatory, anti-complement, anti-thrombotic, anti-diabetic and anti-atherogenic effects [7–10]. In this study, we used an in vivo approach to determine the effect of the plant extract on the immune system of mice.

In the current study, we have found that the *D. morbifera* Léveille extract was shown to increase the proliferation of both T- and B-lymphocytes. The number of splenocytes was increased in mice treated with the plant extract. The rising level of splenic B- and T-cells upon treatment with *D. morbifera* Léveille suggests an immunostimulatory effect of the plant extract on the innate immune system. Interestingly, higher T-cell proliferation was one of the more pronounced effects of plant extract treatment and has been explained by an increase in CD18 phenotypes.

Increasing levels of IFN-γ observed in a dose-dependent manner is indicative of Th1 cell activation by *D. morbifera* Léveille. Th1 immunity is known to defend against cancer development and several intracellular infectious diseases. These results agree with previous reports that suggest the anti-cancer activity of the plant extract [4].

Dose-dependent suppression of IL-4 results in the downregulation of immunoglobulins. These results show suppression of immunoglobulins, which may be an indicator of suppression of Th2 cells or antibody-mediated immunity [16, 17]. There is a chance of reducing purified B lymphocyte IgM and IgG synthesis in stimulated human peripheral blood mononuclear cells (PBMCs) without killing B cells [18]. This indicates the downregulation of the antibody-mediated immune system by the plant extract.

The levels of IL-1α were decreased in the plasma of mice treated with *D. morbifera* Léveille. The proinflammatory cytokine IL-1α is an initiator of immune responses via induction of neutrophil infiltration. Moreover, it stimulates the transcription and secretion of IL-1β, which is a known amplifier of inflammation [19]. Hence, the suppression of these cytokines shows the anti-inflammatory activity of the plant extract. This has been previously indicated in the work of Akram and colleagues [13]. A compound in *D. morbifera* Léveille might inhibit the gene expression of proinflammatory cytokines, such as IL-1α, IL-1β, TNF-α and IL-6, in mice by decreasing COX-2 mRNA expression, as has been shown for the bioactive compound of citrus plants [20].

## Conclusions

This study shows that *D. morbifera* Léveille treatment increases the number of lymphocytes and promotes proliferation of T and B lymphocytes. This can be indicative of boosting non-specific immunity by the plant extract. However, the plant demonstrated suppressive effects in which it inhibited immunoglobulins by inhibiting synthesis/release of IL-4, a known stimulator of antibody-mediated immunity. In addition, the anti-inflammatory effects of the plant were shown through downregulation of the proinflammatory cytokine IL-1β. Although further mechanistic studies on the molecular mechanism of *D. morbifera* Léveille activity are needed, this study has demonstrated immunomodulatory effects of the plant extract in a murine model.

## Abbreviations

CD: Cluster of differentiation
CON-A: concanavalin A
FBS: Fetal bovine serum
HBSS: Hank’s balanced salt solution
IG: Immunoglobulins
IL: Interleukin
LPS: Lipopolysaccharide
PBMC: peripheral blood mononuclear cells
TNF: Tumor necrosis factor
